# Immune-Related RNA-Binding Protein-Based Signature With Predictive and Prognostic Implications in Patients With Lung Adenocarcinoma

**DOI:** 10.3389/fmolb.2022.807622

**Published:** 2022-05-13

**Authors:** Lei Xu, Wanru Li, Ting Yang, Siqi Hu, Qiong Zou, Ju Jiao, Ningyi Jiang, Yong Zhang

**Affiliations:** ^1^ Zhongshan School of Medicine, Sun Yat-sen University, Guangzhou, China; ^2^ Department of Nuclear Medicine, The Seventh Affiliated Hospital, Sun Yat-sen University, Shenzhen, China; ^3^ Department of Nuclear Medicine, The Third Affiliated Hospital, Sun Yat-sen University, Guangzhou, China

**Keywords:** lung adenocarcinoma, immune-related RNA-binding proteins, overall survival, immune microenvironment, cancer immunotherapy

## Abstract

**Background:** Dysregulation of RNA-binding proteins (RBPs) in cancers is associated with immune and cancer development. Here, we aimed to profile immune-related RBPs in lung adenocarcinoma (LUAD) and construct an immune-related RBP signature (IRBPS) to predict the survival and response to immunotherapy.

**Methods:** A correlation analysis was performed to establish a co-expression network of RBPs and immune-related genes (IRGs) to characterize immune-related RBPs in the TCGA–LUAD cohort (*n* = 497 cases). Then, a combination of the Random survival forest (RSF) and Cox regression analysis was performed to screen the RBPs and establish IRBPS. This was followed by independent validation of IRBPS in GSE72094 (*n* = 398 cases), GSE31210, (*n* = 226 cases), and GSE26939 (*n* = 114 cases). Differences between the low- and high-risk groups were compared in terms of gene mutations, tumor mutation burden, tumor-infiltrating lymphocytes, and biomarkers responsive to immunotherapy.

**Results:** DDX56, CTSL, ZC3H12D, and PSMC5 were selected and used to construct IRBPS. The high-risk scores of patients had a significantly worse prognosis in both training and testing cohorts (*p* < 0.0001 and *p* < 0.05, respectively), and they tended to be older and have an advanced TNM stage. Furthermore, IRBPS was a prognostic factor independent of age, gender, smoking history, TNM stage, and EGFR mutation status (*p* = 0.002). In addition, high-risk scores of IRBPS were significantly correlated with tumor-infiltrating lymphocytes (*p* < 0.05). They also had a high level of PD-L1 protein expression (*p* < 0.01), number of neoantigens (*p* < 0.001), and TMB (*p* < 0.001), implying the possible prediction of IRBPS in the immunotherapy of LUAD.

**Conclusion:** The currently established IRBPS encompassing immune-related RBPs might serve as a promising tool to predict survival, reflect the immune microenvironment, and predict the efficacy of immunotherapy among LUAD patients.

## Introduction

Worldwide, 2.2 million new lung cancer cases were diagnosed, and 1.8 million deaths occurred in 2020 (Sung et al., 2021). Although the incidence of lung cancer in the United States has decreased in recent years, it remains the leading cause of cancer deaths, with approximately 350 deaths per day projected by 2022 (Siegel et al., 2022). Non-small-cell lung cancer (NSCLC) accounts for about 85% of all lung cancer cases (Bade and Dela Cruz, 2020). Lung adenocarcinoma (LUAD) is the most common pathological subtype of NSCLC, accounting for about 63% of NSCLC (Gridelli et al., 2015). Although the multidisciplinary cooperative treatment strategy based on traditional treatment methods (surgery, chemotherapy, radiotherapy, and targeted therapy) reduces mortality, the LUAD 5-year overall survival rate remains 15.9% (Chen et al., 2014). Compared with traditional treatment methods, immunotherapy, especially immune checkpoint inhibitors, has changed the treatment pattern of lung cancer. Some patients can obtain a long-lasting treatment response, but this only accounts for 20–40% of all patients (Garon et al., 2019; Brahmer et al., 2018; Article, 2019; Garon et al., 2019; Brahmer et al., 2018; Article, 2019). Based on a high-throughput and micro-assay sequencing development, identifying and recognizing single markers or multifactor markers has become a hotspot in our current research (Ahluwalia et al., 2021; Yi et al., 2021; Zhang et al., 2020a). RNA-binding proteins (RBPs) are critical regulators of posttranscriptional processes (RNA splicing, modification, transport, cell localization, stability, translation, and degradation) (Wang et al., 2018; Martin and Ephrussi, 2009; Moore and Proudfoot, 2009; Fu and Ares, 2014; Martin and Ephrussi, 2009; Moore and Proudfoot, 2009; Fu and Ares, 2014; and Wang et al., 2018) and play an essential role in the occurrence and development of diseases, including cancer (Pereira et al., 2017). Identifying RBPs with potential, predictive, or prognostic functions may further broaden and open up new molecular markers.

Recently, the role of RBPs in the field of cancer has been recognized. The dysregulated expression of certain RBPs can lead to cancer. For example, the mutation of SF3B1 is observed in about 10–15% of patients with chronic lymphocytic leukemia (Wang et al., 2011); overexpression of mRNA 5′cap-binding protein eIF4E promotes the malignant transformation of human and mouse cells (Avdulov et al., 2004). Moreover, there are dozens of RBPs in the currently known cancer driver genes (Zhang et al., 2020b). In addition, some studies have proven that the dysregulation expression of RBPs in cancer relative to adjacent normal tissues is related to the efficacy and prognosis (Wang et al., 2018). For example, overexpression of MEX3B in tumors can reduce the lethality of tumor-infiltrating lymphocytes, thereby mediating tumor immunotherapy resistance (Huang et al., 2018); NONO promotes cancer proliferation by regulating SKP2 and E2F8, significantly affecting patient prognosis (Iino et al., 2020). More importantly, RBPs are considered essential regulators in the immune system (Jeltsch and Heissmeyer, 2016; Turner and Díaz-Muñoz, 2018; Newman et al., 2016; Jeltsch and Heissmeyer, 2016; Newman et al., 2016; and Turner and Díaz-Muñoz, 2018) and may be involved in immune modulation in the tumor microenvironment. However, RBPs associated with immune-related genes (IRGs) are rarely reported.

In this study, by establishing a co-expression network between RBPs and IRGs, we undertook a systematic and comprehensive biomarker discovery and validation effort to identify and develop an IRBPS for robust, prognostic, and efficacious prediction of LUAD patients. Herein, we first report a novel 4-gene immune-infiltrating and proliferation-associated IRBPS that not only identifies patients with poor prognosis but is also unique because of the close association with immune cell infiltration and immunotherapeutic biomarkers. In summary, the IRBPS gene signature provides an attractive platform for risk stratification of prognosis and efficacy in patients with LUAD, which has important implications for the clinical management of patients with this fatal malignancy.

## Methods

### Transcript Data Acquisition and Preprocessing

Genome-wide mRNA expression and clinicopathological information (age, gender, stage, smoking, and follow-up) of the TCGA–LUAD were downloaded as training cohorts from UCSC Xena (https://xenabrowser.net/); 497 LUAD primary tumor tissues matched with the complete follow-up recording were collected. Before further analysis, we converted the quantitative gene expression of fragment per kilobase million (FPKM) into transcripts per million (TPM) values processed by log2(value + 1) for all samples. Gene copy number data of the TCGA–LUAD quantified by the GISTIC2 approach were acquired from the cbioportal database (https://www.cbioportal.org/). Gene-level mutation (VarScan) of the TCGA–LUAD was obtained from the TCGA portal (https://www.cancer.gov/) by the R package “TCGAbiolinks.” The proteomic data of the TCGA-LUAD were downloaded from the Clinical Proteomic Tumor Analysis Consortium (CPTAC) (https://cptac-data-portal.georgetown.edu/). Invalidation sets and normalized transcriptomic data recorded by series matrix files in gene expression series (GSE) including GSE72094, GSE31210, and GSE26939 were downloaded from the Gene Expression Omnibus (GEO) (https://www.ncbi.nlm.nih.gov/geo/), respectively. The probe IDs in the microarray datasets were converted into gene symbols. Only cases with complete follow-up recordings were collected; 6,094 unique candidate RBPs in Homo sapiens were offered from RBP2GO (https://rbp2go.dkfz.de/); 1,793 unique IRGs were extracted from the Immunology Database and Analysis Portal (ImmPort) database (https://www.immport.org/home).

### Identification of Immune-Related RBPs With Prognosis Significance

Significant prognostic RBPs and IRGs in 497 LUAD samples were identified using the univariate Cox proportional-hazards regression analysis, respectively; genes were considered significant at *p* < 0.01. Immune-related RBPs were identified using the Pearson correlation analysis between RBP and IRG expression value. The threshold was set as a *p*-value < 0.01 and |correlation coefficient| > 0.7.

### Construction and Validation of IRBPS at the Transcription Level

The random survival forest (RSF) algorithm is suitable for survival analysis of high-dimensional genomic data by reducing the dimensionality of features. The selection of genes was based on the variable importance (VIMP) and the minimum depth, where both were evaluation indexes to measure the predictive ability of variables for survival prediction. Here, integration of immune-related RBPs into an RFS approach was used to select genes and only choose the intersection of the top 15 genes in both VIMP and minimum depth. Before constructing the Cox regression model, RBPs with a high mutual covariance in the presence of 15 genes were removed by correlation analysis (the cutoff value of *p* < 0.01 and |correlation| > 0.7) to prevent overfitting of the constructed model, and the remaining genes were included in the multivariate analysis. The best model for predicting the prognosis of LUAD patients was determined based on the Akaike information criterion (AIC). The following formula calculated the risk score of each LUAD patient: risk score = expression of gene a * coefficient *a* + expression of gene b * coefficient *ß* + expression of gene c * coefficient *?* + …… + expression of gene n * coefficient n. The median value of the risk scores stratified high- and low-risk groups. The Kaplan–Meier survival curves were applied for survival comparison between low- and high-risk groups, and the log-rank test was used to test statistical significance (*p*-value < 0.05). The performance of the IRBPS was evaluated and validated in the TCGA–LUAD cohorts and GEO–LUAD cohorts, respectively.

### Expression Profile of Genes in IRBPS at the Transcription Level

In the TCGA–LUAD cohort, the Wilcoxon test was used to calculate the differential expression levels of genes in IRBPS between LUAD and normal lung tissues. Copy number variation (CNV) is a necessary part of genome structure variation, leading to variation in gene expression levels. Then, to explore whether the differences in gene expression in IRBPS are affected by CNV, we extracted CNV data defined by the GISTIC2 method and performed a correlation analysis between the gene expression values and CNV values.

### Validation of Expression and Predictive Value of IRBPS at the Protein Level

Protein is the ultimate biological and functional unit for gene function. We separated proteomic data of genes in IRBPS from the Clinical Proteomic Tumor Analysis Consortium (CPTAC, https://proteomics.cancer.gov/programs/cptac) LUAD cohort to verify these gene expression features between LUAD and normal lung tissues. Immunohistochemical staining images were isolated from the Human Protein Atlas project (https://www.proteinatlas.org/) to verify the actual expression of these genes. Furthermore, 111 LUAD cases were obtained with complete follow-up data at an unshared log-ratio-quantified protein expression level. IRBPS at the protein level was also calculated using the aforementioned formula, and multivariate Cox regression analysis identified the independent role of the signature.

### Clinical and Molecular Characteristics Between Low- and High-Risk Groups

The different prognostic characteristics between low- and high-risk groups might demonstrate that these patients have other clinical features and molecular profiles. Therefore, we performed a differential analysis incorporating clinical information such as age, gender, TNM stage, and survival status, as well as carving out the mutational characteristics of patients in different subgroups using the R package “maftools,” all of which were used to assess clinical and genomic differences between groups.

### GO and KEGG Analyses

To further explore the differences in molecular functions and pathways between low- and high-risk groups, the Limma algorithm was used to perform differentially expressed genes (DEGs), with a false discovery rate (FDR) < 0.01 and | log2 (fold change) | > 0.5 as thresholds. Then, the potential biological processes and pathways involved in DEGs were explored through WebGestalt (http://www.webgestalt.org/).

### Profile of Tumor-Infiltrating Immune Cells

In order to explore the relationship between IRBPS and tumor-infiltrating immune cells, the tumor immune score of the TCGA–LUAD cohort was extracted from the Tumor Immune Estimation Resource (TIMER, https://cistrome.shinyapps.io/timer/). The abundance of 24 immune cells in the TCGA–LUAD cohort was obtained from the immune cell abundance identifier (ImmuCellAI, https://cibersort.stanford.edu/). Thus, the immune score and immune cell components between the low- and high-risk groups were compared.

### Exploring the Potential of IRBPS in Immunotherapy Response Prediction

To explore relationships between IRBPS and immunotherapy biomarkers, PD-L1 protein expression, neoantigen, and tumor mutation burden (TMB) data were collected for TCGA–LUAD. PD-L1 protein expression values were extracted from cBioPortal (https://www.cbioportal.org/) calculated by the reverse-phase protein array (RPPA) analysis. TMB and neoantigen datasets were attained from the Cancer Immunome Atlas (TCIA) (https://tcia.at/home). The aforementioned immunotherapy biomarker data were pooled and compared between the low- and high-risk groups.

### Statistical Analysis

The Wilcoxon test and the chi-square test were used in comparisons of the two groups for continuous variables and categorical variables, respectively. All statistical analyses were performed using R software (version 4.0.2). *p* < 0.05 was considered statistically significant.

## Results

### Construction of a Co-Expressed Network Between Selected RBPs and IRGs

Overall, 1,367 RBPs and 194 IRGs were significantly associated with prognosis (*p* < 0.01) in 497 LUAD patients, respectively (Supplementary Tables S1, S2). A two-by-two correlation line analysis of RBPs and IRGs was performed to establish co-expression networks (Supplementary Table S3). Finally, 309 immune-related RBPs were identified based on screening criteria (|correlation| > 0.7 and *p* < 0.01).

### Establishment of IRBPS for Predicting Prognosis in the TCGA–LUAD Cohort

**FIGURE 1:**
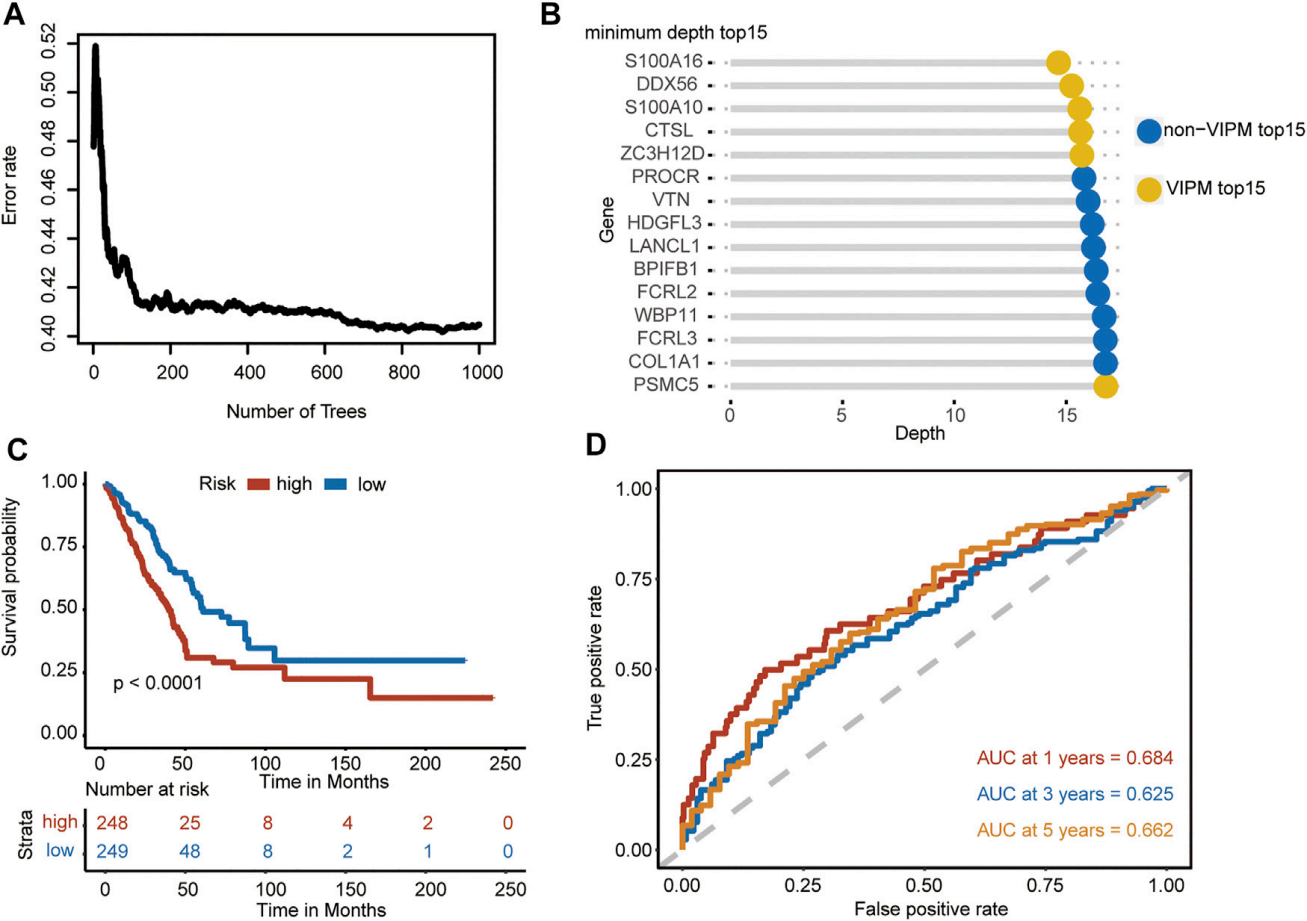
Gene selection and model construction. **(A)** Error plots using the out-of-bag prediction of error estimator based on trees. **(B)** Overlap genes (annotated yellow) ranked by minimum depth and VIPM. **(C)** Kaplan–Meier curves and log-rank test of the overall survival based on the low- and high-risk groups in the TCGA–LUAD. **(D)** ROC curves for evaluating the prediction performance of the signature in the TCGA–LUAD.

309 RBPs were fed into the RFS algorithm. The forest prediction error stabilized as the number of trees increased (Figure 1A). The top 15 RBPs ranked by minimum depth are listed in Figure 1B, where the yellow markers (S100A16, DDX56, S100A10, CTSL, ZC3H12D, and PSMC5) were genes that were also in the top 15 of the VIMP ranking. A correlation analysis between the genes was performed for S100A16, DDX56, S100A10, CTSL, ZC3H12D, and PSMC5, and all genes satisfied mutual independence (Supplementary Figure S1). The stepwise Cox analysis illustrated that the model combining DDX56, CTSL, ZC3H12D, and PSMC5 had the smallest AIC as the best model. Therefore, the multivariate Cox regression model combining four genes was developed as IRBPS. The risk score for each patient was calculated according to the formula: 0.2127 * DDX56 + 0.2453 * CTSL - 0.2487 * ZC3H12D + 0.1431 * PSMC5. Patients were classified into the low- and high-risk groups based on the median risk score as the cutoff point (cutoff value = −0.01). The Kaplan–Meier survival curves demonstrated that patients in the high-risk group had a significantly worse overall survival (OS) in patients with higher risk scores (*p* < 0.0001) (Figure 1C). The area under the ROC curves (AUC) for 1-, 3-, and 5-years was 0.684, 0.625, and 0.662, respectively (Figure 1D). Furthermore, the results of the univariate and multivariate Cox model analyses confirmed that the risk score-based IRBPS was a significant prognostic factor independent of age, gender, smoking history, TNM stage, and EGFR mutation status (HR = 1.63, 95% CI 1.19-2.22, *p* = 0.002) (Table 1).

**TABLE 1:** Univariate and multivariate Cox analysis for prognosis in the TCGA-LUAD cohort.

	Univariate model			Multivariate model		
Variables	HR	CI 95%	P Value	HR	CI 95%	P Value
**Age (years)**	1.01	0.99–1.02	0.305	-	-	-
**Gender**	-	-	-	-	-	-
female	1	-	-	-	-	-
male	0.75	0.78–1.41	0.747	-	-	-
**Stage**	-	-	-	-	-	-
I	1	-	-	1	-	-
II	2.47	1.71–3.57	0.000	2.31	1.60–3.34	0.000
III	3.55	2.42–5.22	0.000	3.19	2.16–4.71	0.000
IV	3.88	2.24–6.75	0.000	3.61	2.07–6.30	0.000
**Smoking**	-	-	-	-	-	-
no	1	-	-	-	-	-
yes	1.05	0.77–1.43	0.747	-	-	-
**EGFR**	-	-	-	-	-	-
wild	1	-	-	-	-	-
mutation	0.69	0.26–1.87	0.472	-	-	-
**Risk Score**	-	-	-	-	-	-
low	1	-	-	1	-	-
high	1.89	1.39–2.55	0.000	1.63	1.19–2.22	0.002

### Validating the Predictive Robustness of IRBPS in Independent Cohorts

**FIGURE 2:**
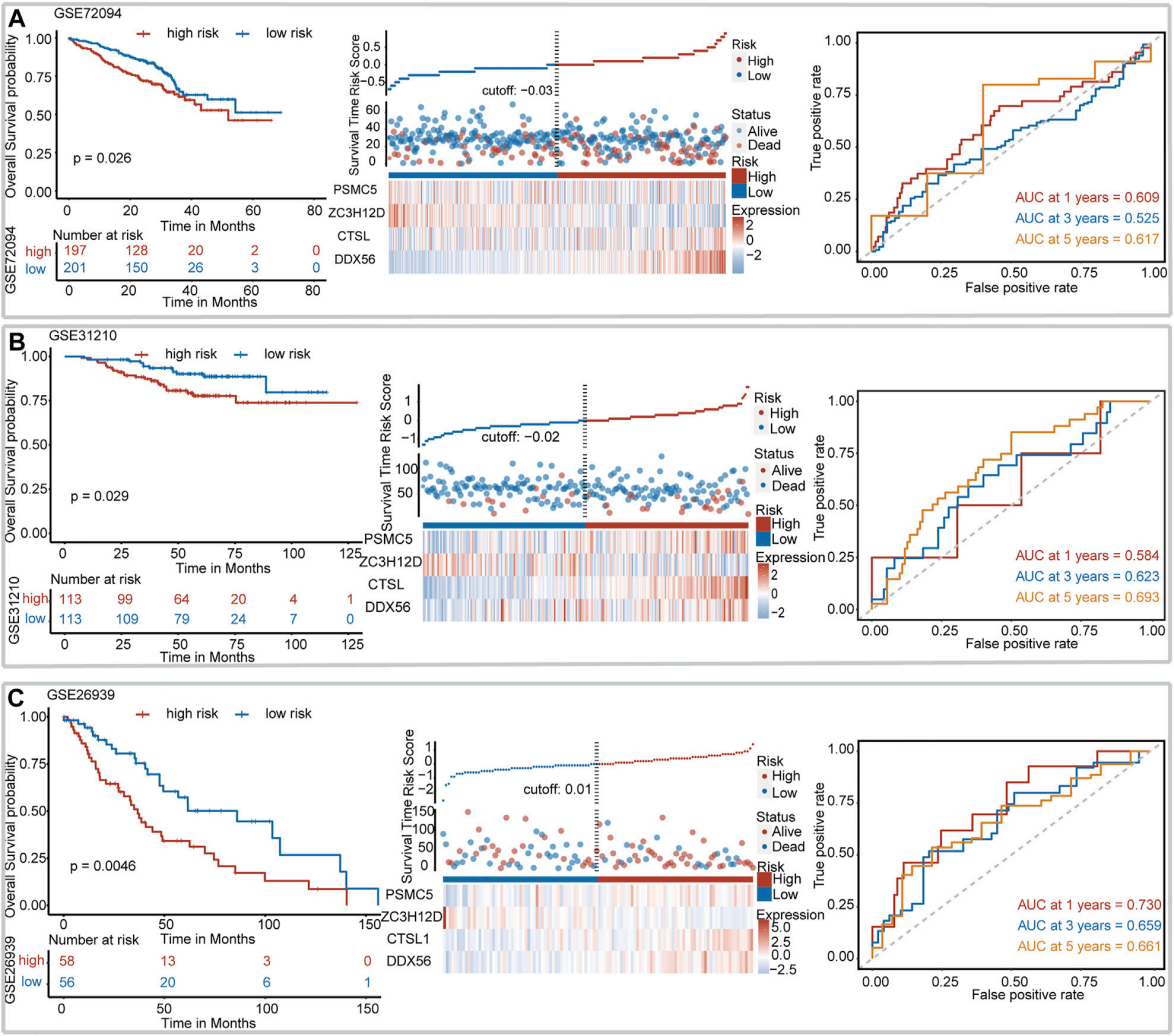
Validation of the signature in independent cohorts. **(A)** GSE72094, **(B)** GSE31210, and **(C)** GSE26939.

We assessed the robustness of IRBPS derived from the TCGA–LUAD cohort in three independent GEO cohorts, including GSE72094, GSE31210, and GSE26939. Patients in these cohorts were classified into low- and high-risk groups using the median risk score cutoffs calculated with the same formula. The results of the Kaplan–Meier curve showed that IRBPS significantly stratified patients in the GSE72094 cohort (cutoff value = −0.03, *p* = 0.026) (Figure 2A), GSE31210 cohort (cutoff value = −0.02, *p* = 0.029) (Figure 2B), and GSE26939 cohort (cutoff value = 0.01, *p* = 0.005) (Figure 2C). AUC values illustrated the good predictive performance of IRBPS in the GSE72094 cohort (0.609, 0.525, and 0.617 for 1-, 3-, and 5-years), GSE31210 cohort (0.584, 0.623, and 0.693 for 1-, 3-, and 5-years), and GSE26939 cohort (0.730, 0.659, and 0.661 for 1-, 3-, and 5-years).

### Expression Profile of Genes in IRBPS at the Transcription Level

**FIGURE 3:**
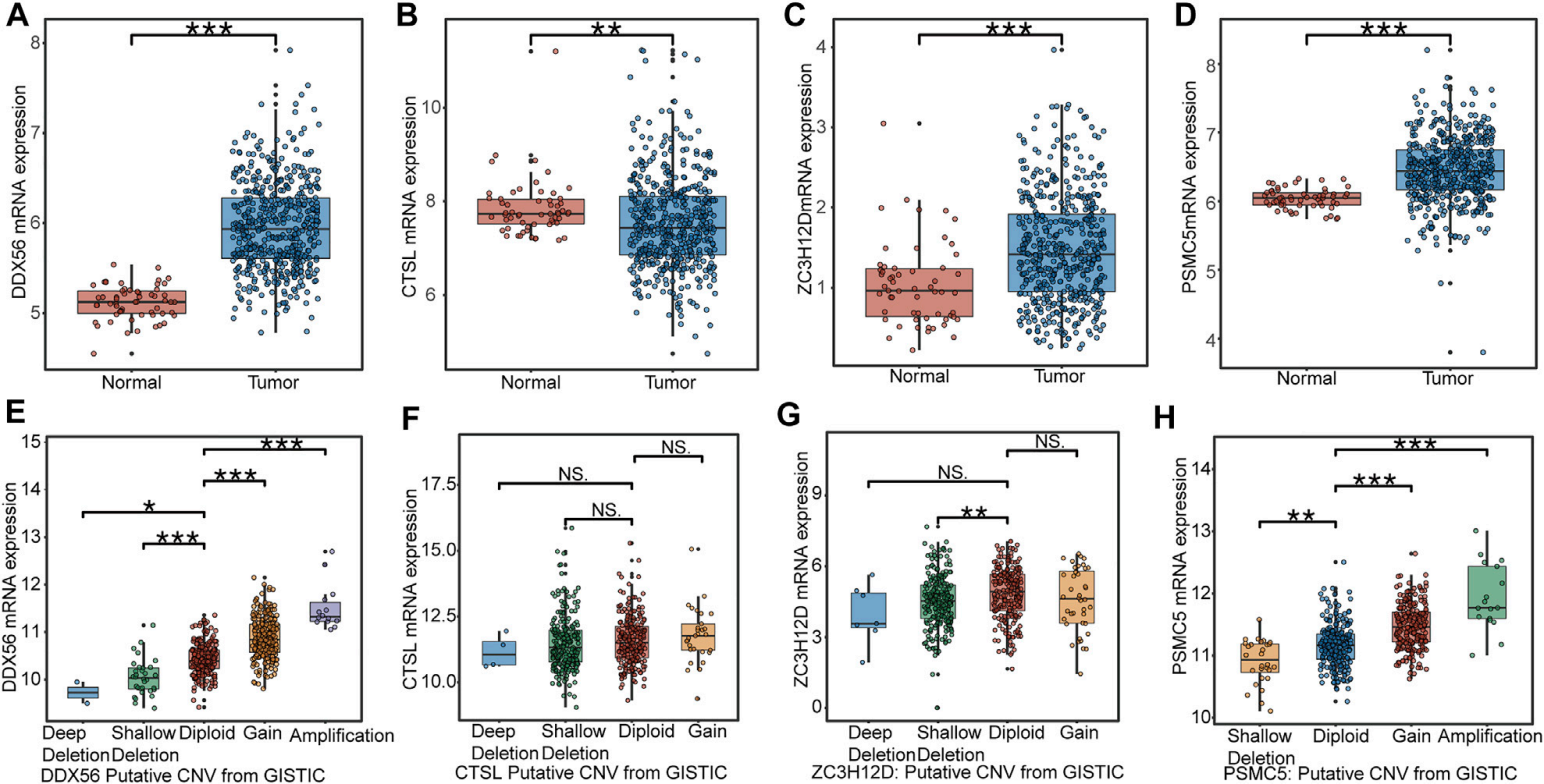
Expression profile of RBPs in IRBPS at the transcription level. **(A–D)** Expression differences between LUAD tissues and normal lung tissues. **(E–H)** The correlation of CNV with the four genes’ expression levels. Diploid implied the absence of CNV at this gene location. Compared with diploid, investigating the effect of other types (deep deletion and shallow deletion gain amplification) of CNV on their gene expression.

We collected 497 LUAD samples and 58 normal lung samples with the transcription data. The expression levels of DDX56 (Figure 3A), ZC3H12D (Figure 3C), and PSMC5 (Figure 3D) were all significantly increased in LUAD tissues compared to normal lung tissues. The CTSL expression had a higher level in normal lung tissues than LUAD tissues (Figure 3B). A diploid implied the absence of CNV at this gene location. Compared with the diploid, other types (deep deletion and shallow deletion gain amplification) of CNV were significantly correlated with gene expressions of DDX56 (Figure 3E), ZC3H12D (Figure 3G), and PSMC5 (Figure 3H) (*p* < 0.05), except for CTSL (Figure 3F) (*p* > 0.05), indicating that CNV might regulate the expression of these genes in cancer.

### Expression Profile of Genes in IRBPS at the Protein Level

**FIGURE 4:**
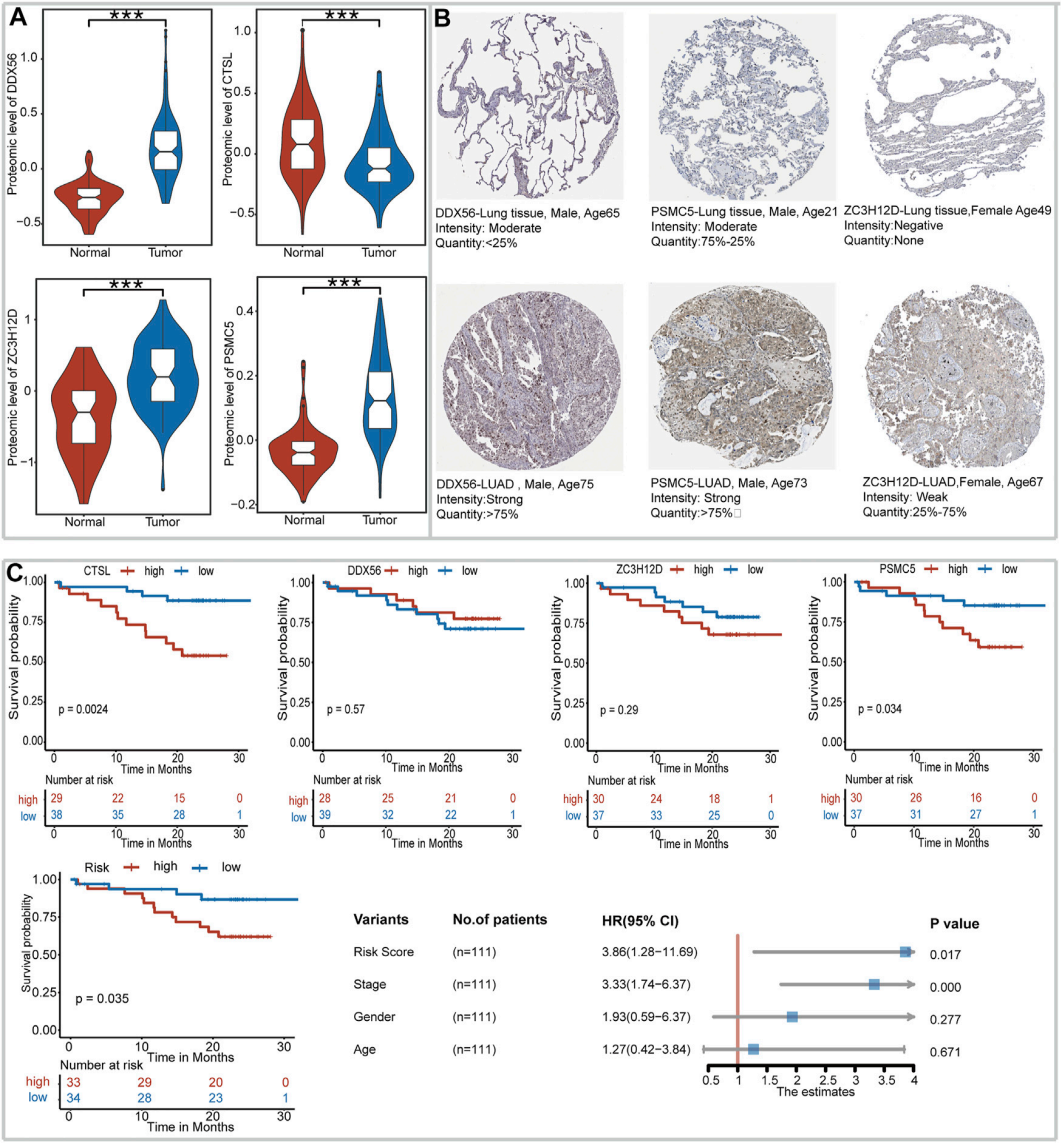
Expression profile of genes in IRBPS at the protein level. **(A)** Gene expression differences between LUAD tissues and normal lung tissues at the protein level. **(B)** Immunohistochemical staining for DDX56, ZC3H12D, and PSMC5 in LUAD tissues and normal lung tissues. **(C)** Kaplan–Meier curves, log-rank test, and multivariate Cox analysis of the overall survival based on the expression level of these genes and IRBPS at the protein level.

At the protein level, proteomic data of 111 LUAD tissues and 106 normal tissues were collected. As shown in Figure 4A, LUAD tissues had a significantly higher expression level of these four genes (DDX56, ZC3H12D, and PSMC5) than normal lung tissues. The CTSL expression had a higher level in normal lung tissues than in LUAD tissues. Immunohistochemical images of the LUAD tissues and normal lung tissues for DDX56, ZC3H12D, and PSMC5 are shown in Figure 4B, respectively. CTSL, due to lack of documentation, was not available for immunohistochemical images. As illustrated in Figure 4C, the high protein level of CTSL and PSMC5 had a significant adverse impact on the survival of LUAD patients. The effect of DDX56 and ZC3H12D on survival did not show statistical significance and might be limited by the sample size. In addition, the high-risk score of IRBPS still adversely affected the survival of LUAD patients and was not affected by age, gender, and TNM stage at the protein level. This further demonstrated the ability of IRBPS for predicting the patient prognosis at the protein level.

### Clinical and Genomic Mutational Features Between IRBPS Subgroups

**FIGURE 5:**
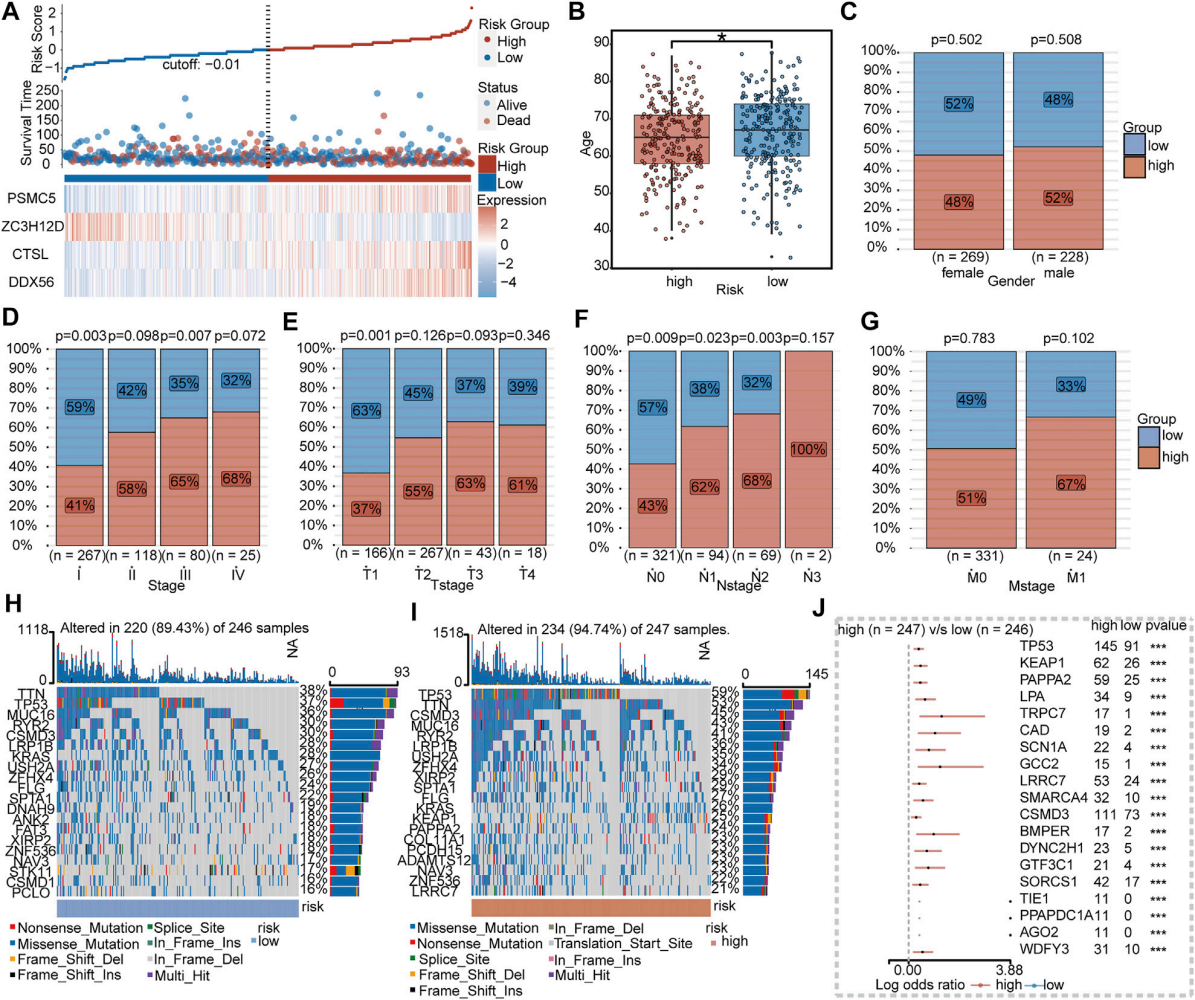
Clinical and mutational features between IRBPS subgroups. **(A)** Distribution of survival status and the four-gene expressions trend between IRBPS subgroups. Clinical characteristic differences between IRBPS subgroups in terms of age **(B)**, gender **(C)**, stage **(D)**, T-stage **(E)**, N-stage **(F)**, and M-stage **(G)**. **(H,I)** Ranked the top 20 of gene mutations in low- and high-risk groups. **(J)** The significant mutation genes in the high-risk group compared to the low-risk group.

As shown in Figures 5A–G, patients in the high-risk group tended to be older and had advanced TNM stage than the low-risk group. The 20 most frequently mutated genes in the low- and high-risk groups were calculated, respectively (Figures 5H,I). By comparing between subgroups, 19 genes including TP53, KEAP1, PAPPA2, LPA, TRPC7, CAD, SCN1A, GCC2, LRRC7, SMARCA4, CSMD3, BMPER, DYNC2H1, GTF3C1, SORCS1, TIE1, PPAPDC1A, AGO2, and WDFY3 were significantly mutated in the high-risk group (Figure 5J).

### Biological Processes Between IRBPS Subgroups

**FIGURE 6:**
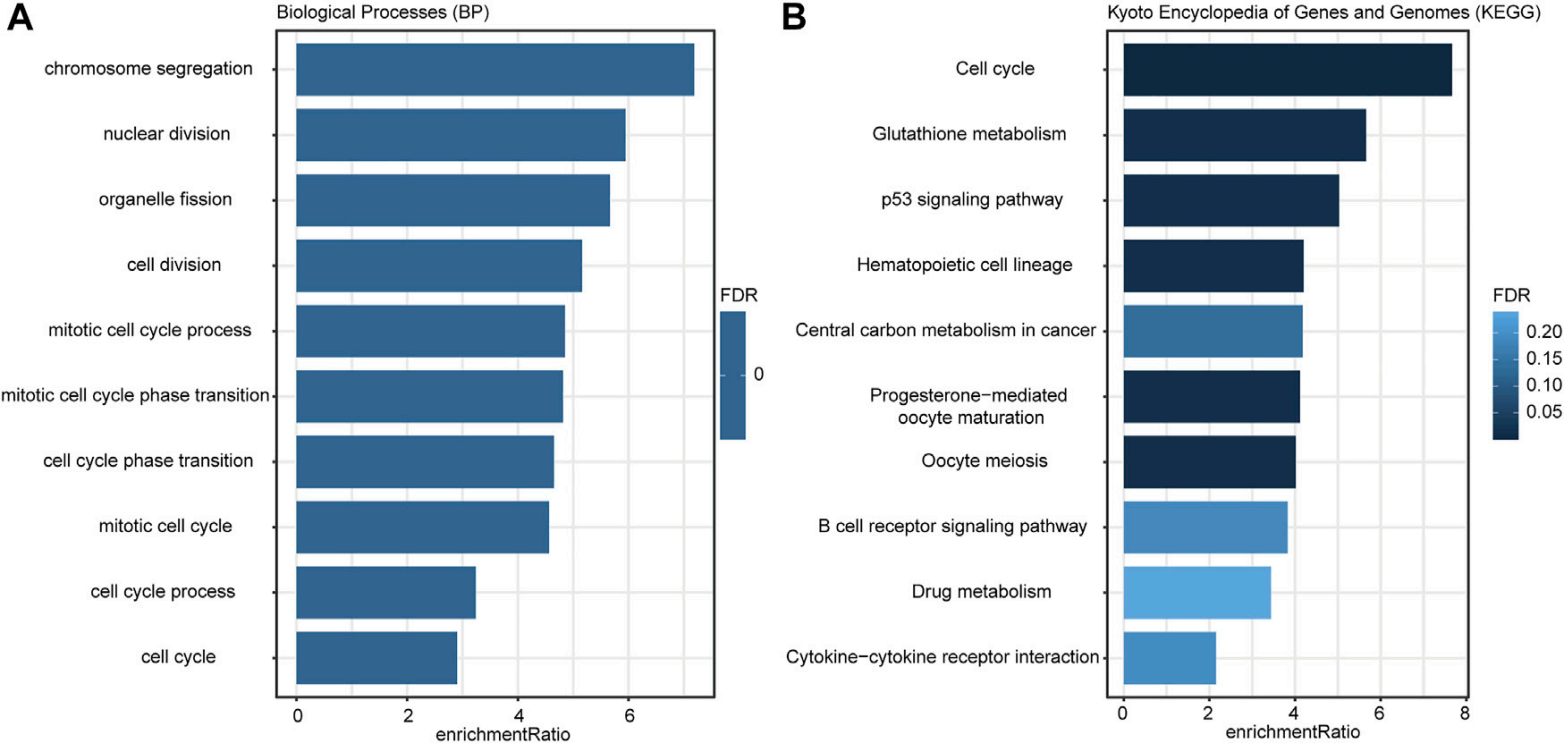
Enrichment results of GO **(A)** and KEGG **(B)** analyses. The color of the bar indicated the significance level; the length of the bar indicated the enrichment ratio.

According to the criteria (FDR <0.01 and | log 2 (Fold Change) | > 0.7), there were 389 DEGs between the low- and high-risk groups (Supplementary Table S3), and they were applied to GO and KEGG analyses. The enrichment results (Figure 6A) indicated that these DEGs were more involved in the biological functions of the regulation of cell proliferation and cell cycle processes. KEGG analysis (Figure 6B) illustrated that these genes were closely associated with cell cycle, metabolism, p53 signaling pathways, and the B-cell reporter signaling pathway.

### Correlation of Tumor-Infiltrating Immune Cells and Immunotherapy Biomarkers With IRBPS Subgroups

**FIGURE 7:**
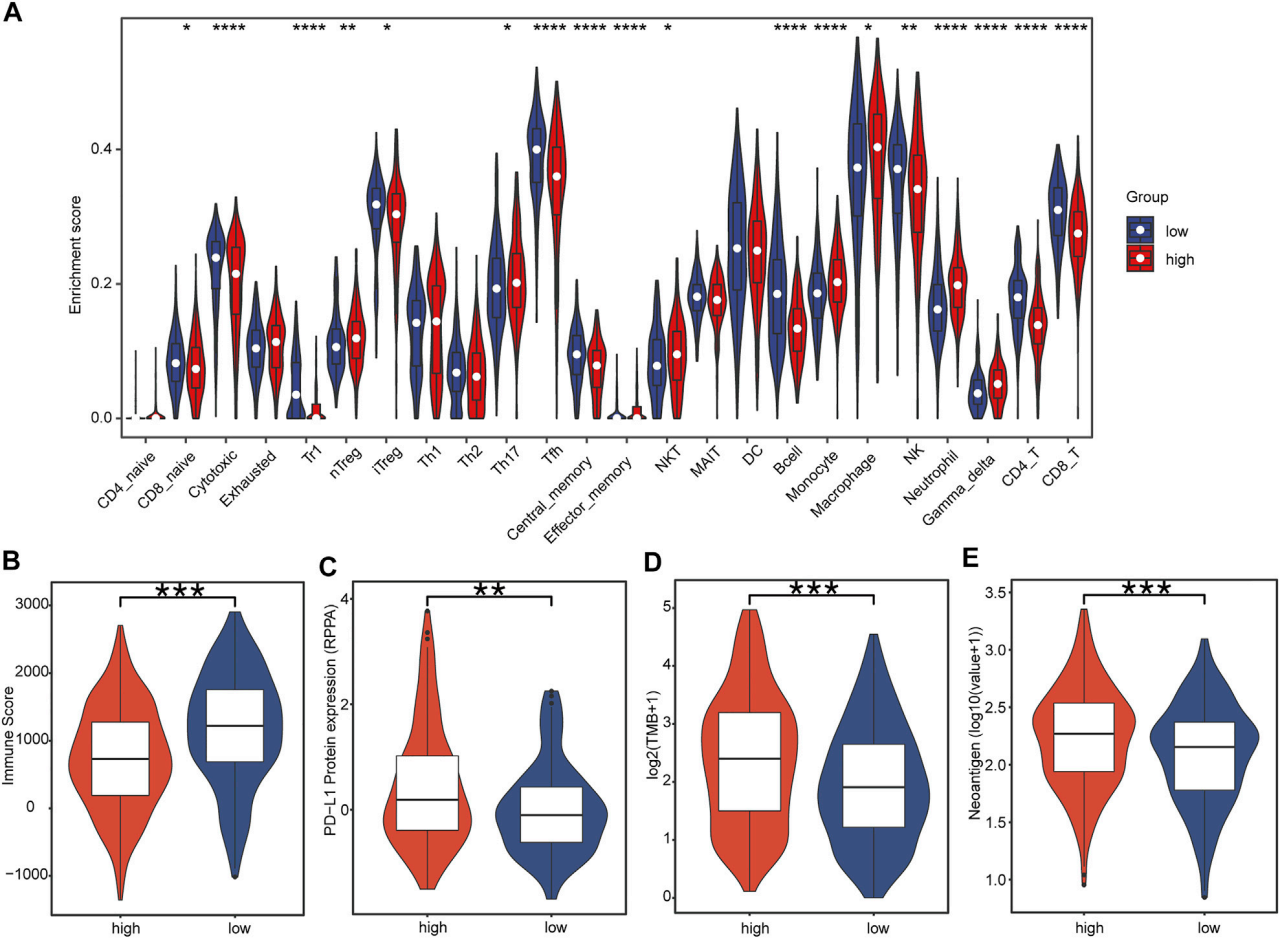
Correlation of tumor-infiltrating immune cells and immunotherapy biomarkers with IRBPS subgroups. **(A)** Abundances of immune cells between subgroups. **(B–E)** Correlation of immunotherapy response biomarkers with subgroups.

Immune cell panorama was significantly different between low- and high-risk patients (*p* < 0.05) (Figure 7A), and the immune score calculated by the TIMER algorithm was significantly higher in the low-risk group (*p* < 0.001) (Figure 7B). As shown in Figure 7A, the abundance of nTreg (*p* = 0.000), Th17 (*p* = 0.018), monocytes (*p* = 0.000), macrophages (*p* = 0.013), and neutrophils (*p* = 0.000) were significantly higher in the high-risk group than those in the low-risk group. On the contrary, the proportion of CD8_naive (*p* = 0.047), cytotoxic (*p* = 0.000), Tfh (*p* = 0.000), central_memory (*p* = 0.000), B cells (*p* = 0.000), NK (*p* < 0.001), CD4_T (*p* = 0.000), and CD8_T (*p* = 0.000) in the low-risk group was significantly higher. In addition, the predictive value of IRBPS for widely recognized immunotherapy biomarkers was further evaluated. The results demonstrated that these biomarkers had a higher level in the high-risk group than in the low-risk group in terms of the PD-L1 protein expression level (Figure 7C, *p* < 0.01), TMB (Figure 7D, *p* < 0.001), and number of neoantigens (Figure 7E, *p* < 0.001).

## Discussion

RBPs are a group of conserved proteins in eukaryotes that play an essential role in co-transcriptional and post-transcriptional gene regulation. RBPs can interact with RNA to form protein–RNA complexes and participate in biological processes such as cell differentiation, proliferation, and cell fate transition. The imbalance of RBPs has been implicated in various diseases. In particular, several studies have demonstrated that RBPs are closely associated with cancer development, and immune and therapeutic responses. However, studies on RBPs, especially immune-related RBPs in LUAD, are still rarely reported. The present study is the first comprehensive analysis of the prognostic impact of 6,094 RBP candidates in LUAD based on a bioinformatics analysis. In addition, by screening immune-related RBP genes, we established a risk score module (IRBPS) based on these genes. We validated their prognostic value at the transcriptional level with four datasets, respectively. Notably, at the protein level, IRBPS still played an essential role in predicting the survival of LUAD patients. Furthermore, IRBPS was closely associated with tumor-infiltrating immune cells and immunotherapeutic biomarkers, which implied that this signature has an essential role in predicting the efficacy of immunotherapy and deserves further investigation.

In this study, RFS was used for variable selection, followed by the traditional Cox regression analysis to construct the model instead of the most commonly used least absolute shrinkage and selection operator. First, RSF is a combinatorial survival tree method that inherits the advantages of random forest, such as noise resistance, over-fitting prevention, and nonlinear correlation processing. It can be used for variable selection for the survival prediction analysis of high-dimensional data. In addition, the RFS algorithm combines two forms of randomization methods: case resampling and variable sub-setting, to prevent the over-fitting problem, make the results more robust, and predict accurately. Then, the combination of the Cox regression analysis provides an exact formula to calculate risk scores, which is convenient for clinical application. In this study, 309 immune-related RBPs were screened out by establishing a co-expression network of RBPs and IRGs, and four RBPs (DDX56, CTSL, ZC3H12D, and PSMC5) were finally identified to construct IRBPS by RSF and Cox regression analysis. The robustness of IRBPS in predicting the prognosis was validated in three independent GEO datasets, respectively. DDX56, ZC3H12D, and PSMC5 were up-regulated in LUAD tissues compared to normal lung tissues. In contrast, CTSL is down-regulated in LUAD tissues. CNV might be involved in the process of regulating the mRNA expression of DDX56, ZC3H12D, and PSMC5. Here, the number of tumor tissues was greater than the number of normal tissues, which had limited utility for assessing differences in gene expression between the two. Therefore, a comparable and sufficient sample size between the two was required. The protein expression features of the four genes between LUAD tissues and normal lung tissues followed the same trend at the transcription level. Although the CPTAC–LUAD data recorded the protein expression values of four RBPs in 111 patients, there were many missing values of the ZC3H12D gene. We here filled in the missing values by the KNN method to initially assess the prognostic impact of each gene and IRBPS at the protein level. The results showed that high expression of CTSL and PSMC5 still negatively affected the prognosis of LUAD patients, and that IRBPS was an adverse prognostic factor independent of age, gender, and the TNM stage. These results directly or indirectly provide evidence that RBPs are dysregulated in LUAD and affect the prognosis of LUAD patients. Notably, patients in the high-risk group of IRBPS exhibited older age, more advanced tumor stages, had more genetic mutations, lower immune scores, and higher levels of immune efficacy marker indices. This also explains, in one way, why the prognosis is worse in the high-risk group of IRBPS, and it also suggests that patients with high-risk scores of IRBPS may have a higher possibility of immunotherapeutic efficacy.

Previously, many studies have shown that RBPs are associated with immune responses and immune regulation. In the innate immune response, some RBPs (such as TTP, Roquin, and Regnase-1) promote inflammation by eliminating the mRNA of pro-inflammatory cytokines (Mino and Takeuchi, 2018), controlling the adaptive immune responses, and maintaining immune homeostasis during T-cell activation (Jeltsch and Heissmeyer, 2016). This study found that IRBPS was significantly associated with the tumor immune score (TIMER) and immune cell infiltration (ImmuCellAI). Based on these results, we explored the potential of IRBPS in predicting the immune response. The results showed higher TMB, PD-L1 protein expression, and the number of tumor neoantigens in the high-risk group than in the low-risk group. These indicators are well-established biological biomarkers for predicting immunotherapeutic efficacy in LUAD. DDX56 encodes a member of the DEAD-box protein family. The pathway analysis showed that DDX56 regulated the p53 signaling pathway, affected cell cycle progression, and promoted cancer cell proliferation (Zhu et al., 2020). The high expression level of the DDX56 gene was correlated with a high level of TP53 mutation and was positively correlated with the MYC signal (Zhu et al., 2020; Cui et al., 2021). MYC could up-regulate PD-L1 protein by interacting with PD-L1 enhancer genes (Casey et al., 2016). TP53 mutation also promoted the PD-L1 protein expression (Cha et al., 2016; Dong et al., 2017). CTSL promoted tumor angiogenesis by regulating the CDP/Cux/VEGF-D pathway (Pan et al., 2020a). VEGF was the most important angiogenesis factor, which inhibited antigen presentation, promoted regulatory T cell infiltration, and induced PD-L1 expression on tumor-infiltrating T cells (Voron et al., 2015). ZC3H12D was a tumor suppressor gene associated with memory T lymphocytes and macrophages, involved in the regulation of inflammation (Wang et al., 2007; Huang et al., 2012). Previous studies showed that ZC3H12D was associated with a variety of immune-related genes, including CD274 (PD-L1) and CTLA4 (Chen et al., 2022). ZC3H12D could regulate IL-6 expression, and IL-6 was positively correlated with the PD-L1 expression (Wawro et al., 2017; Chan et al., 2019). IL-6 enhanced the glycosylation initiation and stability of PD-L1, which was necessary to maintain the stability of PD-L1 and its interaction with PD-1 (Chan et al., 2019). PSMC5 not only has the proteasome function but also participates in transcriptional regulation. PSMC5 regulated the epithelial–mesenchymal transition, hypoxia, immune response, and other pathways, and was positively correlated with gene expression such as CD274 and CTLA4 (He et al., 2021). Obviously, high expression of the four genes (DDX56, CTSL, ZC3H12D, and PSMC5) could promote the expression of PD-L1 as they have been proven to be promising biomarkers and immunotherapy targets in a variety of tumors, including colorectal cancer (Kouyama et al., 2019; He et al., 2021), NSCLC (Wu et al., 2021; Chen et al., 2022), and gastric cancer (Pan et al., 2020b).

Although sufficient verification was achieved in the TCGA–LUAD database and the GEO–LUAD database, the performance of IRBPS in predicting the survival status was still limited (AUC<0.7). IRBPS combined with clinical features to jointly build the model might further enhance the predictive power of the model. Second, this study’s incorporation of multi-omics data was limited to the TCGA portal, which prevented us from extensively validating the robustness of IRBPS. Furthermore, the absence of transcriptome data from patients receiving immunotherapy prevented further evaluation of the potential of IRBPS to predict its immune response in the real world. In addition, future cellular and animal experiments were also warranted to prove this concept in this study. Despite such limitations, it is undeniable that our study provides important clues to elucidate the prognostic value of RBPs in LUAD, to facilitate the selection of beneficiary groups for immunotherapy, and even to develop new therapeutic strategies for patients with LUAD.

In summary, this study first comprehensively characterizes immune-related RBPs with a prognostic value in LUAD and has constructed an IRBPS which offers an easy tool for identifying LUAD patients with worse prognosis and reflecting the efficacy of immunotherapy patients with this lethal malignancy.

## Data Availability

The original contributions presented in the study are included in the article/Supplementary Material; further inquiries can be directed to the corresponding authors.

## Abbreviations

AIC, Akaike information criterion; CPTAC, Clinical Proteomic Tumor Analysis Consortium; CNV, copy number variation; DEG, differentially expressed gene; FDR, false discovery rate; FPKM, fragment per kilobase million; GEO, Gene Expression Omnibus; GSE, gene expression series; IRGs, immune-related genes; IRBPS, immune-related RNA-binding proteins; LUAD, lung adenocarcinoma; OS, overall survival; RBPs, RNA-binding Proteins; RSF, random survival forest; TMB, tumor mutation burden; TCIA, the Cancer Immunome Atlas; TCGA, the Cancer Genome Atlas Program; TPM, transcripts per million.

## References

[B1] Ahluwalia P. Ahluwalia M. Mondal A. K. Sahajpal N. Kota V. Rojiani M. V. (2021). Immunogenomic Gene Signature of Cell-Death Associated Genes with Prognostic Implications in Lung Cancer. Cancers (Basel) 13 (1), 1–18. 10.3390/cancers13010155 PMC7795632 33466402

[B2] Article O. (2019). Five-year Survival Outcomes for Patients with Advanced Melanoma Treated with Pembrolizumab in Original Article. Ann. Oncol. 30 (4), 582–588. 30715153 10.1093/annonc/mdz011 PMC6503622

[B3] Avdulov S. Li S. Van Michalek V. Burrichter D. Peterson M. Perlman D. M. (2004). Activation of Translation Complex eIF4F Is Essential for the Genesis and Maintenance of the Malignant Phenotype in Human Mammary Epithelial Cells. Cancer Cell 5 (6), 553–563. 10.1016/j.ccr.2004.05.024 15193258

[B4] Bade B. C. Dela Cruz C. S. (2020). Lung Cancer 2020: Epidemiology, Etiology, and Prevention. Clin. Chest Med. 41, 1–24. 10.1016/j.ccm.2019.10.001 32008623

[B5] Brahmer J. R. Govindan R. Anders R. A. Antonia S. J. Sagorsky S. Davies M. J. (2018). The Society for Immunotherapy of Cancer Consensus Statement on Immunotherapy for the Treatment of Non-small Cell Lung Cancer (NSCLC). J. Immunotherapy Cancer 6 (1), 75. 10.1186/s40425-018-0382-2 PMC6048854 30012210

[B6] Casey S. C. Tong L. Li Y. Do R. Walz S. Fitzgerald K. N. (2016). MYC Regulates the Antitumor Immune Response through CD47 and PD-L1. Science 352 (6282), 227–231. 10.1126/science.aac9935 26966191 PMC4940030

[B7] Cha Y. J. Kim H. R. Lee C. Y. Cho B. C. Shim H. S. (2016). Clinicopathological and Prognostic Significance of Programmed Cell Death Ligand-1 Expression in Lung Adenocarcinoma and its Relationship with P53 Status. Lung Cancer 97, 73–80. 10.1016/j.lungcan.2016.05.001 27237031

[B8] Chan L.-C. Li C.-W. Xia W. Hsu J.-M. Lee H.-H. Cha J.-H. (2019). IL-6/JAK1 Pathway Drives PD-L1 Y112 Phosphorylation to Promote Cancer Immune Evasion. J. Clin. Invest. 129 (8), 3324–3338. 10.1172/jci126022 31305264 PMC6668668

[B9] Chen W. Guo Z. Wu J. Lin G. Chen S. Lin Q. (2022). Identification of a ZC3H12D-Regulated Competing Endogenous RNA Network for Prognosis of Lung Adenocarcinoma at Single-Cell Level. BMC Cancer 22, 115. 10.1186/s12885-021-08992-1 35090416 PMC8796579

[B10] Chen Z. Fillmore C. M. Hammerman P. S. Kim C. F. Wong K.-K. (2014). Non-small-cell Lung Cancers: a Heterogeneous Set of Diseases. Nat. Rev. Cancer 14, 535–546. 10.1038/nrc3775 25056707 PMC5712844

[B11] Cui Y. Hunt A. Li Z. Birkin E. Lane J. Ruge F. (2021). Lead DEAD/H Box Helicase Biomarkers with the Therapeutic Potential Identified by Integrated Bioinformatic Approaches in Lung Cancer. Comput. Struct. Biotechnol. J. 19, 261–278. 10.1016/j.csbj.2020.12.007 33425256 PMC7779375

[B12] Dong Z.-Y. Zhong W.-Z. Zhang X.-C. Su J. Xie Z. Liu S.-Y. (2017). Potential Predictive Value of TP53 and KRAS Mutation Status for Response to PD-1 Blockade Immunotherapy in Lung Adenocarcinoma. Clin. Cancer Res. 23 (12), 3012–3024. 10.1158/1078-0432.ccr-16-2554 28039262

[B13] Fu X.-D. Ares M. (2014). Context-dependent Control of Alternative Splicing by RNA-Binding Proteins. Nat. Rev. Genet. 15 (10), 689–701. 10.1038/nrg3778 25112293 PMC4440546

[B14] Garon E. B. Hellmann M. D. Rizvi N. A. Carcereny E. Leighl N. B. Ahn M. J. (2019). Five-Year Overall Survival for Patients with Advanced Non‒Small-Cell Lung Cancer Treated with Pembrolizumab: Results from the Phase I KEYNOTE-001 Study. J. Clin. Oncol. 37 (28), 2518–2527. 10.1200/JCO.19.00934 31154919 PMC6768611

[B15] Gridelli C. Rossi A. Carbone D. P. Guarize J. Karachaliou N. Mok T. (2015). Non-small-cell Lung Cancer, Nat. Rev. Dis. Primers, 1. 15009. 10.1038/nrdp.2015.9 27188576

[B16] He Z. Yang X. Huang L. Zhou L. Zhang S. Sun J. (2021). PSMC5 Promotes Proliferation and Metastasis of Colorectal Cancer by Activating Epithelial-Mesenchymal Transition Signaling and Modulating Immune Infiltrating Cells. Front. Cel Dev. Biol. 9, 657917. 10.3389/fcell.2021.657917 PMC8323717 34336824

[B17] Huang L. Malu S. McKenzie J. A. Andrews M. C. Talukder A. H. Tieu T. (2018). The RNA-Binding Protein MEX3B Mediates Resistance to Cancer Immunotherapy by Downregulating HLA-A Expression. Clin. Cancer Res. 24 (14), 3366–3376. 10.1158/1078-0432.ccr-17-2483 29496759 PMC9872773

[B18] Huang S. Qi D. Liang J. Miao R. Minagawa K. Quinn T. (2012). The Putative Tumor Suppressor Zc3h12d Modulates Toll-like Receptor Signaling in Macrophages. Cell Signal 24 (3), 569–576. 10.1016/j.cellsig.2011.10.011 22036805 PMC3237786

[B19] Iino K. Mitobe Y. Ikeda K. Takayama K. i. Suzuki T. Kawabata H. (2020). RNA‐binding Protein NONO Promotes Breast Cancer Proliferation by post‐transcriptional Regulation of SKP2 and E2F8. Cancer Sci. 111 (1), 148–159. 10.1111/cas.14240 31733123 PMC6942431

[B20] Jeltsch K. M. Heissmeyer V. (2016). Regulation of T Cell Signaling and Autoimmunity by RNA-Binding Proteins. Curr. Opin. Immunol. Elsevier Ltd 39, 127–135. 10.1016/j.coi.2016.01.011 26871597

[B21] Kouyama Y. Masuda T. Fujii A. Ogawa Y. Sato K. Tobo T. (2019). Oncogenic Splicing Abnormalities Induced by DEAD ‐Box Helicase 56 Amplification in Colorectal Cancer. Cancer Sci. 110 (10), 3132–3144. 10.1111/cas.14163 31390121 PMC6778637

[B22] Martin K. C. Ephrussi A. (2009). mRNA Localization: Gene Expression in the Spatial Dimension. Cell 136, 719–730. 10.1016/j.cell.2009.01.044 19239891 PMC2819924

[B23] Mino T. Takeuchi O. (2018). Post-transcriptional Regulation of Immune Responses by RNA Binding Proteins. Proc. Jpn. Acad. Ser. B: Phys. Biol. Sci. 94 (6), 248–258. 10.2183/pjab.94.017 PMC6085518 29887569

[B24] Moore M. J. Proudfoot N. J. (2009). Pre-mRNA Processing Reaches Back toTranscription and Ahead to Translation. Cell 136, 688–700. 10.1016/j.cell.2009.02.001 19239889

[B25] Newman R. McHugh J. Turner M. (2016). RNA Binding Proteins as Regulators of Immune Cell Biology. Clin. Exp. Immunol. 183 (1), 37–49. 10.1111/cei.12684 26201441 PMC4687516

[B26] Pan T. Jin Z. Yu Z. Wu X. Chang X. Fan Z. (2020). Cathepsin L Promotes Angiogenesis by Regulating the CDP/Cux/VEGF-D Pathway in Human Gastric Cancer. Gastric Cancer 23 (6), 974–987. 10.1007/s10120-020-01080-6 32388635 PMC7567730

[B27] Pan T. Jin Z. Yu Z. Wu X. Chang X. Fan Z. (2020). Cathepsin L Promotes Angiogenesis by Regulating the CDP/Cux/VEGF-D Pathway in Human Gastric Cancer. Gastric Cancer 23 (6), 974–987. 10.1007/s10120-020-01080-6 32388635 PMC7567730

[B28] Pereira B. Billaud M. Almeida R. (2017). RNA-binding Proteins in Cancer: Old Players and New Actors. Trends Cancer 3 (7), 506–528. 10.1016/j.trecan.2017.05.003 28718405

[B29] Siegel R. L. Miller K. D. Fuchs H. E. Jemal A. (2022). Cancer Statistics, 2022. CA A. Cancer J. Clinicians 72 (1), 7–33. 10.3322/caac.21708 35020204

[B30] Sung H. Ferlay J. Siegel R. L. Laversanne M. Soerjomataram I. Jemal A. (2021). Global Cancer Statistics 2020: GLOBOCAN Estimates of Incidence and Mortality Worldwide for 36 Cancers in 185 Countries. CA A. Cancer J. Clin. 71 (3), 209–249. 10.3322/caac.21660 33538338

[B31] Turner M. Díaz-Muñoz M. D. (2018). RNA-binding Proteins Control Gene Expression and Cell Fate in the Immune System. Nat. Immunol. 19, 120–129. 10.1038/s41590-017-0028-4 29348497

[B32] Voron T. Colussi O. Marcheteau E. Pernot S. Nizard M. Pointet A. L. (2015). VEGF-A Modulates Expression of Inhibitory Checkpoints on CD8+ T Cells in Tumors. J. Exp. Med. 212 (2), 139–148. 10.1084/jem.20140559 25601652 PMC4322048

[B33] Wang L. Lawrence M. S. Wan Y. Stojanov P. Sougnez C. Stevenson K. (2011). SF3B1 and Other Novel Cancer Genes in Chronic Lymphocytic Leukemia. N. Engl. J. Med. 365 (1), 2497–2506. 10.1056/NEJMoa1109016 22150006 PMC3685413

[B34] Wang M. Vikis H. G. Wang Y. Jia D. Wang D. Bierut L. J. (2007). Identification of a Novel Tumor Suppressor Gene P34 on Human Chromosome 6q25.1. Cancer Res. 67 (1), 93–99. 10.1158/0008-5472.can-06-2723 17210687 PMC3461257

[B35] Wang Z.-L. Li B. Luo Y.-X. Lin Q. Liu S.-R. Zhang X.-Q. (2018). Comprehensive Genomic Characterization of RNA-Binding Proteins across Human Cancers. Cel Rep. 22 (1), 286–298. 10.1016/j.celrep.2017.12.035 29298429

[B36] Wawro M. Kochan J. Krzanik S. Jura J. Kasza A. (2017). Intact NYN/PIN-Like Domain Is Crucial for the Degradation of Inflammation-Related Transcripts by ZC3H12D. J. Cel. Biochem. 118 (3), 487–498. 10.1002/jcb.25665 27472830

[B37] Wu Q. Luo X. Terp M. G. Li Q. Li Y. Shen L. (2021). DDX56 Modulates post-transcriptional Wnt Signaling through miRNAs and Is Associated with Early Recurrence in Squamous Cell Lung Carcinoma. Mol. Cancer 20 (1), 1–17. 10.1186/s12943-021-01403-w 34446021 PMC8393456

[B38] Yi M. Li A. Zhou L. Chu Q. Luo S. Wu K. (2021). Immune Signature-Based Risk Stratification and Prediction of Immune Checkpoint Inhibitor's Efficacy for Lung Adenocarcinoma. Cancer Immunol. Immunother. 70 (79), 1705–1719. 10.1007/s00262-020-02817-z 33386920 PMC8139885

[B39] Zhang B. Babu K. R. Lim C. Y. Kwok Z. H. Li J. Zhou S. (2020). A Comprehensive Expression Landscape of RNA-Binding Proteins (RBPs) across 16 Human Cancer Types. RNA Biol. 17 (2), 211–226. 10.1080/15476286.2019.1673657 31607220 PMC6973330

[B40] Zhang Y. Yang M. Ng D. M. Haleem M. Yi T. Hu S. (2020). Multi-omics Data Analyses Construct TME and Identify the Immune-Related Prognosis Signatures in Human LUAD. Mol. Ther. - Nucleic Acids 21, 860–873. 10.1016/j.omtn.2020.07.024 32805489 PMC7452010

[B41] Zhu C. Zhang X. Kourkoumelis N. Shen Y. Huang W. (2020). Integrated Analysis of DEAD-Box Helicase 56: A Potential Oncogene in Osteosarcoma. Front. Bioeng. Biotechnol. 8 (June), 588. 10.3389/fbioe.2020.00588 32671031 PMC7332757

