# Supplementary figures and images for: Immune-Related RNA-Binding Protein-Based Signature With Predictive and Prognostic Implications in Patients With Lung Adenocarcinoma
Source: Front Mol Biosci. 2022 May 13;9:807622. doi: 10.3389/fmolb.2022.807622 (PMC9136055; doi:10.3389/fmolb.2022.807622)

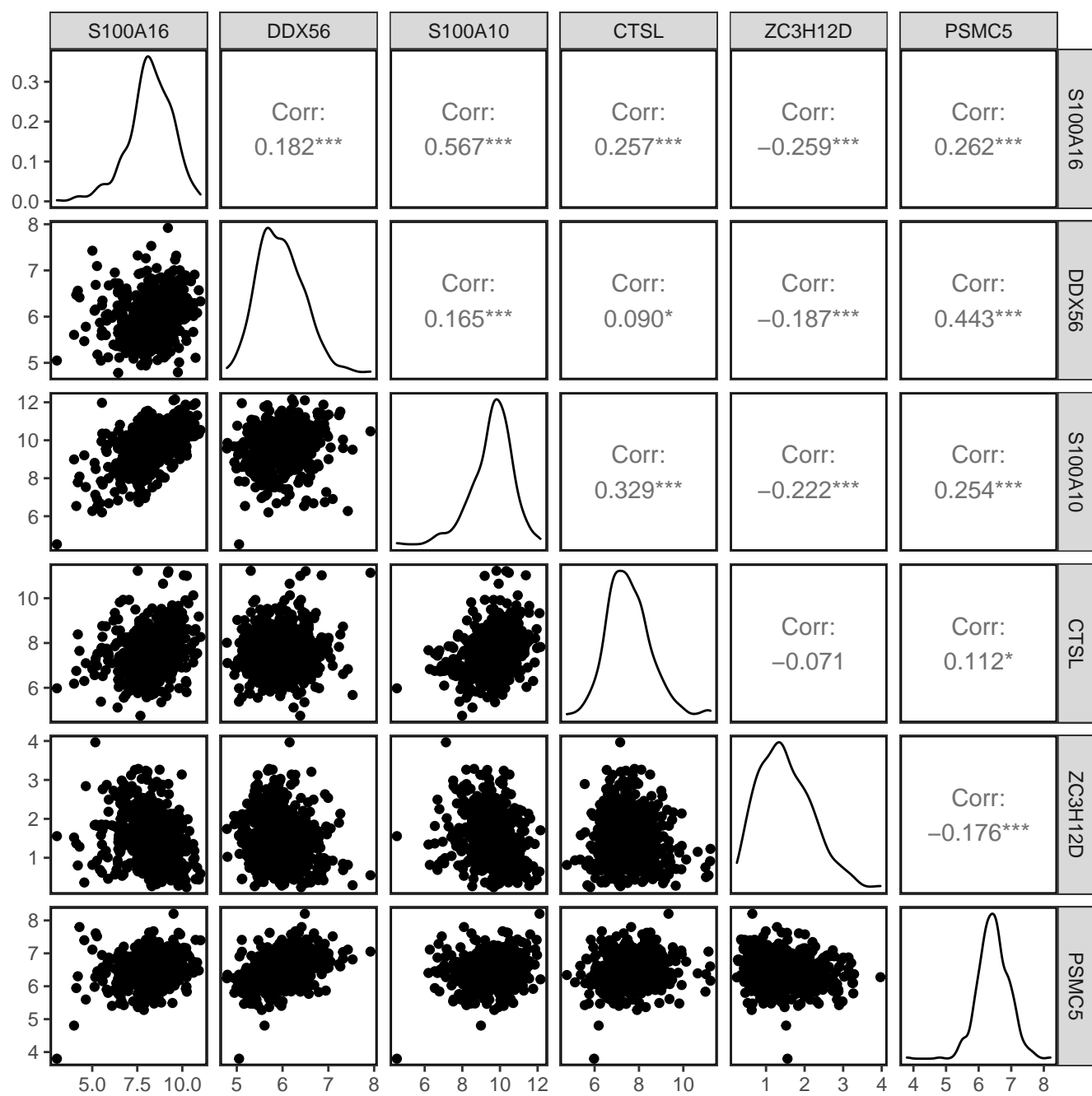

Supplement: Supplementary file 5 [file Image1.pdf]
